# Delayed giant thoracic pseudomeningocele presenting as enlarging painless paraspinal swelling followed by postural dizziness: a case report

**DOI:** 10.3389/fsurg.2026.1954854

**Published:** 2026-09-30

**Authors:** Zhi-jun Lu, Chao-yang Guo, Fei-xiang Lin

**Affiliations:** 1 Department of Spine Surgery, Ganzhou People’s Hospital, Ganzhou, Jiangxi, China; 2 Department of Spine Surgery, Ganzhou Hospital-Nanfang Hospital, Southern Medical University, Ganzhou, Jiangxi, China; 3 Department of Spine Surgery, Affiliated Ganzhou Hospital, Jiangxi Medical College, Nanchang University, Ganzhou, Jiangxi, China

**Keywords:** epidural cyst, leakage of cerebrospinal fluid, postural dizziness, pseudomeningocele, thoracic ossification of the ligamentum flavum

## Abstract

**Introduction:**

Pseudomeningocele (PMC) is an uncommon but recognized complication of spinal surgery. In this report, we present a rare case of delayed thoracic PMC presenting as a progressively enlarging, painless paraspinal swelling followed by postural dizziness, occurring 8 months after thoracic laminectomy performed for thoracic ossification of the ligamentum flavum (tOLF).

**Case presentation:**

A 46-year-old woman underwent T9–L1 laminectomy with posterior instrumentation and fusion for tOLF associated with incomplete paraplegia. During the initial surgery, no dural tear or CSF leakage was identified, including during the Valsalva manoeuvre. MRI at 3 months showed no evidence of PMC. At 8 months postoperatively, she developed a progressively enlarging, painless, fluctuant thoracic paraspinal mass followed by postural dizziness, without postural headache or signs of infection. Cranial MRI showed no evidence of spontaneous intracranial hypotension (SIH), and cardiac and carotid examinations revealed no cause for the dizziness. Revision surgery confirmed a PMC with a 2.0 mm × 2.0 mm dural defect, which was repaired using a dural patch with reinforced fascial closure. At 1-year follow-up, the PMC and postural dizziness had completely resolved, with no evidence of recurrence.

**Conclusion:**

We present an uncommon case of delayed thoracic PMC presenting with an enlarging, painless paraspinal swelling followed by postural dizziness, but without postural headache, 8 months after spinal decompression and fusion for tOLF. Follow-up MRI at 3 months after surgery had shown no evidence of PMC, and there was no antecedent trauma. Cranial MRI showed no imaging evidence of SIH. MRI plays a crucial role in establishing the diagnosis. Given the recognised association between tOLF and intraoperative dural tears, delayed PMC should be considered when a painless, progressively enlarging paraspinal swelling develops after surgery for tOLF, even in the absence of postural headache, worsening paraparesis, or imaging evidence of SIH.

## 1 Introduction

PMC is a rare complication following spinal surgery. Giant PMC, defined as a lesion exceeding 8 cm in diameter, was first described by Hyndman and Gerber in 1946, with a reported incidence of less than 2% (1, 2). Although PMC may present with various symptoms, including headache, local pain, neurological deficits, and infection, postural dizziness associated with PMC is extremely rare. Here, we report a rare case of delayed giant thoracic PMC, presenting as a progressively enlarging, painless paraspinal swelling followed by postural dizziness, in the absence of postural headache, imaging evidence of SIH, or infection. A 46-year-old woman underwent T9–L1 laminectomy with posterior instrumentation and fusion for tOLF associated with incomplete paraplegia. Eight months postoperatively, without any antecedent trauma or intervention, she developed a progressively enlarging, painless thoracic dorsal paraspinal mass followed by postural dizziness. The dizziness occurred in both standing and supine positions and improved after approximately 5 min of standing or on assuming the lateral decubitus position. Plain radiographs demonstrated T9–L1 pedicle screw–rod instrumentation and absence of the laminae. MRI revealed a large epidural cystic lesion and revision surgery confirmed the diagnosis of PMC. The cyst wall was removed, and a dural defect was identified and repaired using a dural patch. Meticulous multilayer closure of the fascia and paraspinal muscles was performed to eliminate dead space. At 1-year follow-up, the PMC and postural dizziness had completely resolved without recurrence. Giant PMC with postural dizziness is an extremely rare complication following thoracic laminectomy for tOLF. Watertight dural repair combined with reinforced multilayer closure and dead-space elimination may achieve satisfactory outcomes. This case provides clinical insights into the diagnosis and surgical management of this rare postoperative complication.

## 2 Case presentation

A 46-year-old woman with incomplete paraplegia of both lower extremities but no bowel or bladder dysfunction underwent T9–L1 laminectomy with posterior instrumentation and fusion for tOLF. Following surgery, the patient underwent monthly follow-up, during which she received structured rehabilitation guidance aimed at improving lower-extremity motor function and ambulation. Throughout the first 3 postoperative months, she reported no back discomfort, swelling, or local signs suggestive of infection, such as erythema, warmth, or pain. At the 3-month follow-up, MRI demonstrated adequate spinal cord decompression with no evidence of PMC. From 3 to 8 months postoperatively, the patient reported no significant discomfort or other notable symptoms. At 8 months after surgery, she developed a painless, fluctuant, protruding mass at the thoracic surgical site, without an impulse on coughing. The mass gradually enlarged and stabilised in size approximately 11 months postoperatively. No local pain or tenderness was noted upon palpation. The patient also developed postural dizziness, which occurred during abrupt postural changes, including sudden standing and the supine position. The symptoms were relieved after standing for approximately 5 min or in the lateral decubitus position. Notably, sustained compression of the dorsal mass for more than 1 min could reproduce the dizziness. The patient reported increasing concern regarding the prominent thoracic dorsal mass because of its cosmetic impact. In addition, the associated dizziness substantially impaired her ability to maintain prolonged standing or sitting positions and to perform postural changes, thereby interfering with her daily and occupational activities. These symptoms prompted her readmission for further evaluation and treatment. She was a manual worker with two children. There was no known family history of tOLF among her parents, sibling, or children.

Physical examination revealed a 16-cm longitudinal surgical scar over the thoracic dorsal region and a localized protruding mass measuring approximately 17 cm × 10 cm (Figure 1). The mass was fluctuant on palpation, with normal overlying skin color and temperature. No skin ulceration, sinus tract, or fluid discharge was observed. There was no obvious muscle atrophy of the bilateral buttocks or lower extremities. Muscle tone of both lower limbs was normal. Manual muscle testing demonstrated grade 5 strength in bilateral hip flexion and grade 4 strength in bilateral knee extension, ankle dorsiflexion, plantar flexion, and great toe extension. Bilateral patellar and Achilles tendon reflexes were hyperactive. Bilateral ankle clonus and Babinski signs were positive.

**Figure 1:**
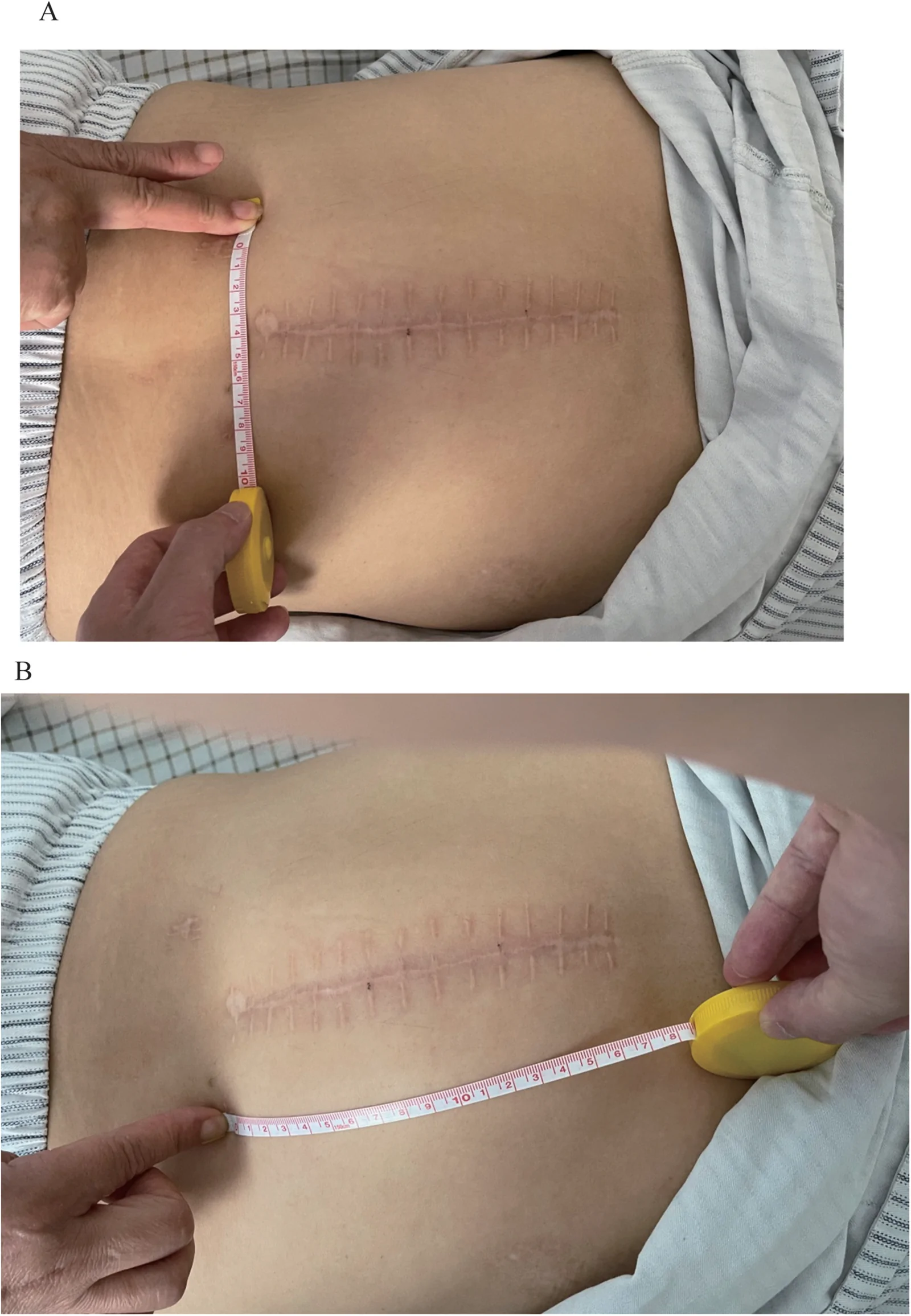
A giant bulging mass measuring approximately 17 cm × 10 cm was observed in the thoracic dorsal region **(A,B)**.

Further evaluation, including cranial MRI, electrocardiography, echocardiography, and carotid artery ultrasonography, revealed no abnormalities associated with the patient's dizziness. Cranial MRI showed no imaging evidence of SIH, with no subdural collections, pachymeningeal enhancement, venous engorgement, pituitary enlargement, brain sagging, or cerebellar tonsillar descent. Laboratory examinations, including complete blood count, erythrocyte sedimentation rate, C-reactive protein, and procalcitonin levels, were within normal limits. Plain radiographs demonstrated posterior instrumentation with pedicle screws and rods extending from T9 to L1, along with absence of the laminae at these levels. Thoracic MRI revealed a large subcutaneous cystic lesion in the dorsal region measuring approximately 14.3 cm × 9.0 cm × 5.0 cm. The fluid signal within the cyst was continuous with the cerebrospinal fluid (CSF) signal in the dural sac, suggesting communication between the cystic cavity and the intradural space. Thoracolumbar MRI demonstrated no spinal cord tissue or abnormal spinal cord signal within the cystic lesion and no evidence of mural nodules (Figure 2). As contrast-enhanced MRI was not performed, enhancement of the PMC wall could not be assessed. Based on the patient's clinical presentation, laboratory findings, and imaging characteristics, postoperative infection and other postoperative fluid collections were considered unlikely. Furthermore, cranial MRI and carotid ultrasonography revealed no intracranial pathology or carotid vascular abnormalities that could account for her dizziness. Collectively, these findings supported a preliminary diagnosis of postoperative PMC. Given the substantial size of the lesion and the need to directly identify the suspected dural defect and confirm communication between the cystic cavity and the subarachnoid space, surgical exploration was performed to establish the definitive diagnosis.

**Figure 2:**
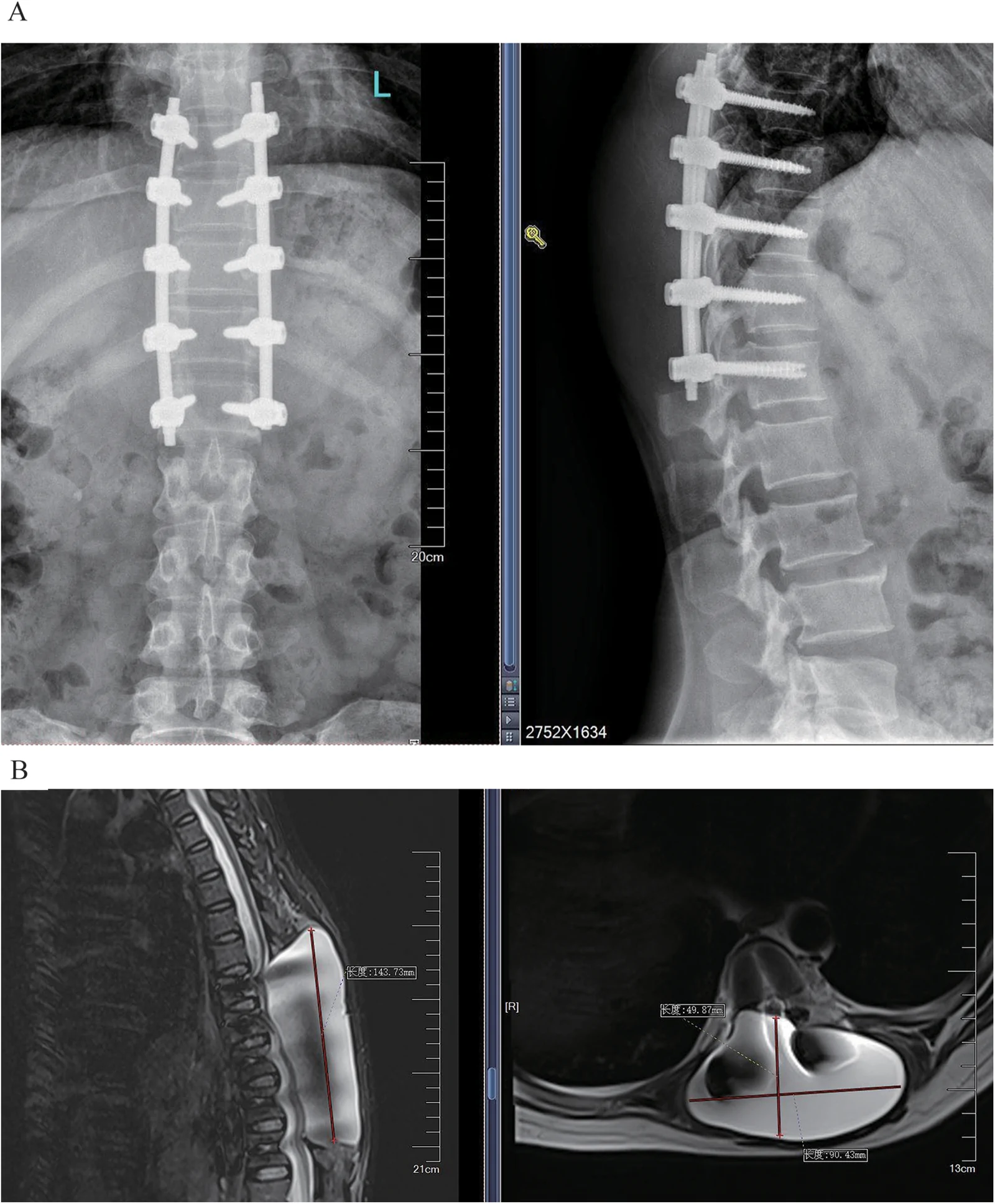
Anteroposterior and lateral radiographs of the thoracic spine demonstrating absence of the T9–L1 laminae and posterior pedicle screw–rod instrumentation **(A)** T2-weighted MRI of the thoracic spine demonstrating a giant epidural cyst **(B)**.

The patient underwent surgical resection of the cystic lesion. During the procedure, the patient was placed in the prone position. Considering the possibility of communication between the cyst and the dural sac, we were concerned that sudden release of the cystic fluid could result in rapid CSF loss, acute intracranial hypotension, and potentially life-threatening complications, including brain herniation and cardiorespiratory instability. Therefore, before opening the cyst wall, approximately 100 mL of clear, pale yellow fluid was carefully aspirated from the cyst. The patient was closely monitored for 5 min, and vital signs remained stable. The cyst was then opened, revealing a well-formed fibrous cyst wall. A 2.0 mm × 2.0 mm dural defect was identified on the right side of the dorsal aspect of the dural sac at the proximal end of the laminectomy site, directly communicating with the cystic cavity. The cyst wall was completely excised, and the dural defect was repaired using a dural patch (Figure 3). A 22-mm diameter silicone drainage tube was placed in the epidural space. Subsequently, meticulous multilayer closure of the fascia, paraspinal muscles, soft tissues, and skin was performed to achieve watertight closure and eliminate potential dead space.

**Figure 3:**
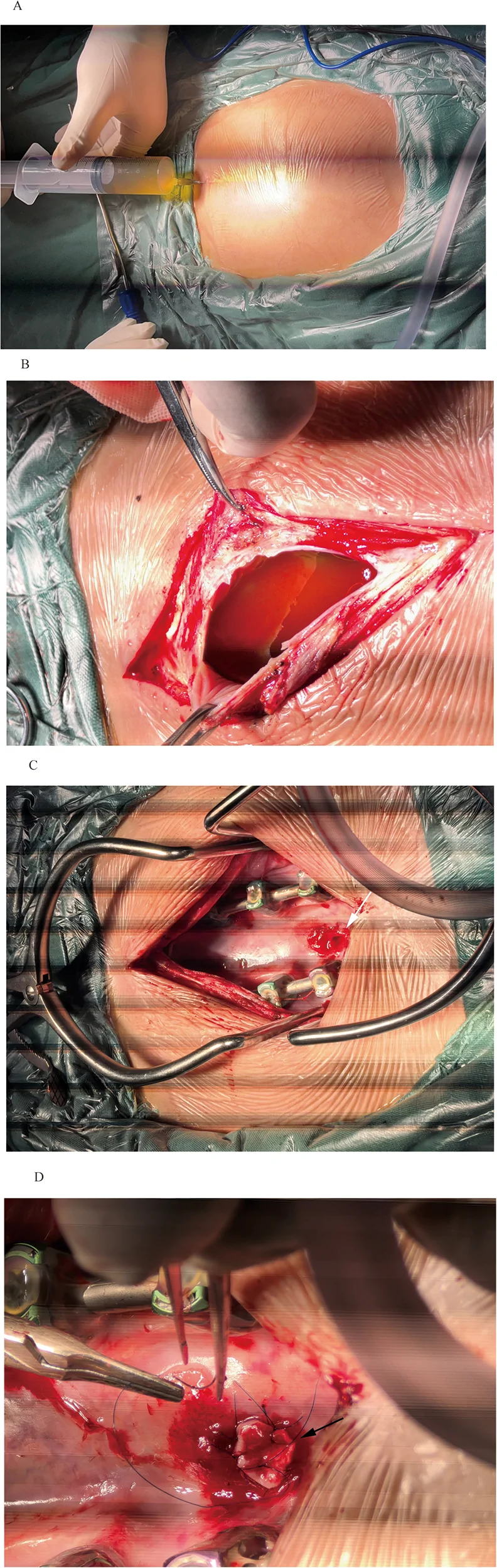
Aspiration of approximately 100 mL of clear, pale-yellow fluid from the cyst before cyst incision **(A)** intraoperative photographs showing the cyst filled with clear, light yellow fluid **(B)** the white arrow indicates the dural defect **(C)** the black arrow indicates the dural patch used to repair the dural defect **(D)**.

After emergence recovery from anesthesia, the patient's bilateral lower-extremity muscle strength and sensory function remained unchanged compared with the preoperative status. The surgical site was covered with compressive dressings, and the patient was instructed to rest in the lateral decubitus or prone position with the head lower than the feet while hospitalized. The color and volume of the drainage fluid were monitored daily.

On postoperative day 7, the drainage volume was approximately 10 mL within 24 h, and the drainage tube was removed. On postoperative day 9, the patient was allowed to ambulate, after which the postural dizziness completely resolved. The protruding mass in the thoracic dorsal region disappeared. During surgery, fluid aspirated from the PMC was submitted for laboratory analysis. The fluid analysis was consistent with CSF, and both aerobic and anaerobic bacterial cultures were negative. At 1 month after the second surgery, thoracolumbar MRI revealed a small amount of residual fluid within the thoracic dorsal soft tissues without evidence of cyst formation. During the 1-year follow-up, the thoracic dorsal mass and postural dizziness completely resolved without recurrence. The patient was satisfied with the surgical outcome, resumed her normal activities of daily living, and returned to her previous occupation as a manual worker. At the 1-year follow-up, physical examination revealed a flat thoracic dorsal contour with normal skin color and temperature and no palpable fluctuance; compression of the surgical site did not provoke dizziness. Motor strength was 5/5 for bilateral hip flexion and 4/5 for bilateral knee extension, ankle dorsiflexion, plantar flexion, and great-toe extension. Bilateral patellar and Achilles tendon reflexes remained hyperreflexic, with positive ankle clonus and Babinski signs bilaterally. MRI at 1 year confirmed complete resolution of the PMC without recurrence (Figure 4). The patient's clinical course is summarized in Table 1. Long-term follow-up will continue to monitor for recurrence of the PMC and postural dizziness, as well as changes in neurological function.

**Figure 4:**
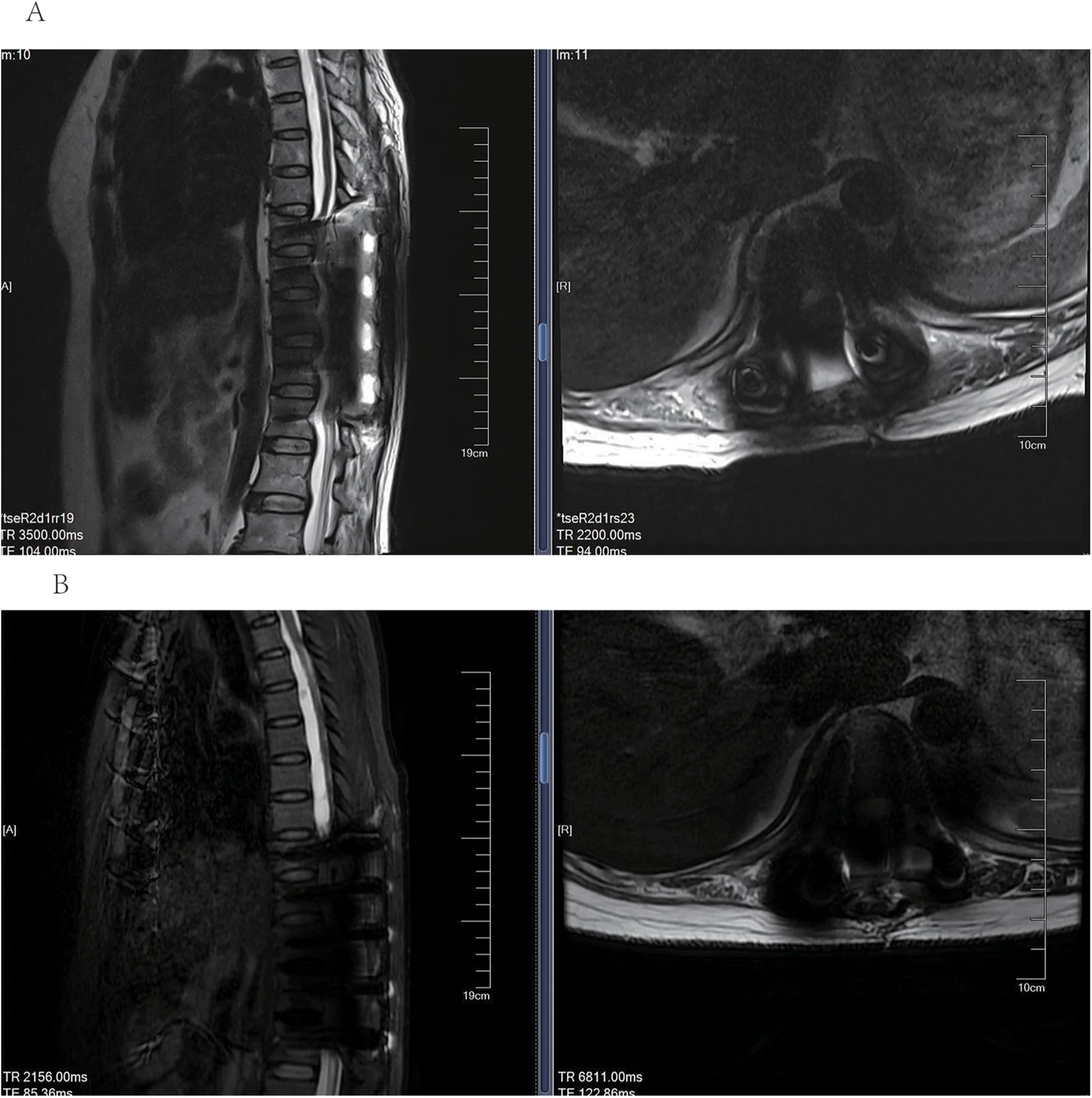
T2-weighted MRI of the thoracic spine 3 weeks after surgery demonstrating complete resolution of the giant PMC with minimal residual soft-tissue fluid collection **(A)** T2-weighted MRI obtained 2 months after surgery demonstrating complete resolution of the residual soft-tissue fluid collection **(B)**.

**Table 1:** Timeline of the patient's clinical course.

Time	Clinical course and evaluation	Management and outcome
Initial presentation	TOLF associated with incomplete paraplegia of both lower extremities.	T9-L1 laminectomy with posterior instrumentation and fusion.
1–8 months after the first surgery	Monthly follow-up; no back discomfort, swelling, erythema, warmth, or pain. MRI at 3 months demonstrated adequate spinal cord decompression.	Rehabilitation guidance was provided to improve lower-extremity motor and ambulatory function.
8 months after the first surgery	A thoracic dorsal mass was first noted. The patient also experienced mild postural dizziness, which did not interfere with her daily activities.	Continued follow-up.
11 months after the first surgery	The thoracic dorsal mass had stabilized in size, whereas postural dizziness had worsened and interfered with prolonged standing or sitting, postural changes, and daily and occupational activities.	Readmission for further evaluation and treatment.
Readmission	Cranial MRI, electrocardiography, echocardiography, and carotid ultrasonography revealed no abnormalities explaining the dizziness. Inflammatory markers were within normal limits. Thoracic MRI demonstrated a large cystic lesion measuring approximately 14.3 cm × 9.0 cm × 5.0 cm, with imaging findings suggestive of communication with the subarachnoid space.	Postoperative PMC was considered the preliminary diagnosis.
Second surgery	Surgical exploration revealed a 2 mm × 2 mm dural defect on the right dorsal aspect of the dural sac at the proximal end of the laminectomy site, directly communicating with the cystic cavity.	Surgical treatment of the PMC was performed.
1 month after the second surgery	MRI demonstrated a small amount of residual fluid in the thoracic dorsal soft tissues without cyst formation.	Continued follow-up.
1 year after the second surgery	The thoracic dorsal mass and postural dizziness had completely resolved. MRI confirmed complete resolution of the PMC without recurrence.	The patient resumed normal activities of daily living and returned to her previous occupation as a manual worker

## 3 Discussion

PMC is an uncommon but recognized complication of spinal surgery. PMC is primarily associated with congenital, traumatic, or iatrogenic dural defects. CSF leaks through the dural defect into the surrounding epidural soft tissues while maintaining communication with the subarachnoid space, eventually forming of a fibrous cystic cavity (3). Ana Rodrigues and Reza Ehsanian each reported a case of dizziness caused by postoperative PMC following lumbar and cervical decompressive laminectomy, respectively (4, 5). However, to the best of our knowledge, no previous reports have described postoperative thoracic PMC presenting with dizziness. The currently proposed mechanism underlying PMC formation involves continuous CSF leakage into paraspinal soft tissues, resulting in a one-way valve mechanism. In the present case, MRI revealed no evidence of spinal cord herniation, supporting the hypothesis that a valve-like mechanism caused the symptoms. Positional changes may have induced rapid redistribution of a large volume of CSF from the extradural cystic cavity back into the subarachnoid space, leading to abrupt fluctuations in intracranial pressure and transient reduction in cerebral perfusion, thereby resulting in dizziness (6).

PMC may present with a variety of clinical manifestations, including back pain, headache, vomiting, neurological deficits, and, in severe cases, infection. The differential diagnosis of postoperative PMC primarily includes other postoperative fluid collections, particularly seroma, hematoma, and abscess. MRI is the preferred noninvasive imaging modality for the diagnosis and evaluation of PMC, allowing clear delineation of the cystic cavity and assessment of associated spinal cord or neural tissue herniation. It also plays a central role in differentiating PMC from other postoperative fluid collections. When conventional MRI findings are inconclusive, CT myelography may provide additional diagnostic information by demonstrating communication between the fluid collection and the subarachnoid space, thereby helping to confirm or exclude PMC (7). Treatment strategies should be tailored according to the severity of symptoms. Definitive management can be achieved through surgical repair of the dural defect, reinforced multilayer closure of the paraspinal muscles and fascia, and elimination of dead space to prevent persistent CSF leakage (1, 8, 9).

In the present case, the cystic cavity was directly communicating with the dural sac. To avoid sudden CSF loss after cyst opening, which could result in acute intracranial hypotension and potentially life-threatening complications such as brain herniation or cardiorespiratory instability, partial aspiration of the cystic fluid was performed before opening the cyst wall. This procedure was intended to gradually reduce intracystic pressure and allow close monitoring for any adverse effects caused by abrupt changes in CSF pressure. Because the thoracic spinal cord is considered more vulnerable than other spinal regions due to its anatomical characteristics and relatively limited vascular supply, early surgical intervention should be considered once a giant thoracic PMC is diagnosed to prevent progressive neurological deterioration.

This case is subject to certain limitations. We report a patient with a giant PMC and postural symptoms following decompressive laminectomy, whose symptoms completely resolved after surgical treatment. Although alterations in cerebrospinal fluid dynamics, including a possible valve-like mechanism, may provide a plausible pathophysiological explanation for the postural symptoms, no direct evidence was obtained to confirm this mechanism. Nevertheless, the favorable clinical outcome highlights the importance of early recognition of symptomatic PMC and consideration of definitive surgical treatment when clinically indicated. Further studies involving additional cases and more comprehensive imaging and physiological assessments are needed to clarify the underlying mechanisms.

## 4 Conclusions

PMC associated with postural dizziness without headache is an uncommon delayed complication following spinal surgery. Surgery for tOLF is associated with a recognised risk of intraoperative dural tears and CSF leakage. MRI plays a crucial role in establishing the diagnosis when a painless, progressively enlarging paraspinal swelling develops in this setting. In patients presenting with postoperative symptoms such as dizziness, headache, or vomiting, clinicians should consider alterations in intracranial pressure caused by dynamic changes in PMC pressure and CSF circulation. Complete excision of the PMC combined with meticulous repair of the dural defect can effectively resolve the lesion and associated symptoms. This case provides valuable clinical insights into the diagnosis and surgical management of postoperative PMC and highlights the importance of considering this rare complication in patients with unexplained postural symptoms after spinal surgery.

## Data Availability

The original contributions presented in the study are included in the article/Supplementary Material, further inquiries can be directed to the corresponding author.
